# Invasive Group B Streptococcal Infection with Toxic Shock-Like Syndrome in a Postsplenectomy Patient

**DOI:** 10.1155/2020/4048610

**Published:** 2020-02-13

**Authors:** Pradeep Kumar Mada, Gabriel Castano, Andrew Stevenson Joel Chandranesan

**Affiliations:** ^1^Infectious Diseases Department, Louisiana State University Health Sciences Center-Shreveport, Shreveport, LA, USA; ^2^Internal Medicine Department, Texas Health Presbyterian Hospital Dallas, Dallas, TX, USA; ^3^Internal Medicine Department, Louisiana State University Health Sciences Center-Shreveport, Shreveport, LA, USA

## Abstract

The incidence of invasive group B streptococcal disease (GBS) in nonpregnant population is increasing. As per the Centers for Disease Control and Prevention (CDC), there are 10 cases in every 100,000 nonpregnant adults each year, and 1 in 20 nonpregnant adults with serious GBS infections die. GBS infection is almost always associated with underlying risk factors such as diabetes mellitus or malignancy. We present a 47-year-old female with a remote history of splenectomy presented with toxic shock-like syndrome secondary to invasive GBS infection.

## 1. Background

The incidence rates of invasive group B streptococcal disease (GBS) in adults are on the rise. More than two thirds of invasive GBS infections in the United States of America now occurs in nonpregnant adults with a mean age of 60 years and has an associated mortality rate of 20–25% [1]. The common risk factors associated with invasive GBS are advanced age, diabetes mellitus, immunodeficiency conditions, and splenectomy. GBS can be invasive in patients with no identifiable risk factors in up to 24% of cases [2].

## 2. Case Presentation

A 47-year-old white female was transferred to our intensive care unit (ICU) from another hospital for higher level of care. The patient initially presented to the other hospital with nausea, vomiting, diarrhea, and abdominal pain which started three to four hours after consuming a salad consisted of spring mix vegetables, oranges, and balsamic vinegar. Her symptoms progressively worsened over 12 hours, and she was admitted to the other hospital. Her past medical history was significant for pancreatic tail cysts for which she underwent partial pancreatectomy and splenectomy, 12 years prior to the current admission. She denied history of diabetes mellitus, hypertension, malignancy, or genitourinary disorders. She also denied intravenous drug use, tick/mosquito bites, or camping/trekking. Her last menstrual period was 6 weeks prior to the admission. No recent tampon use, no abortions, and no previous menstrual problems were reported.

On admission, the patient was found to be hypotensive (54/21 mm of Hg) with hypothermia (95.3°F) and sinus tachycardia (109/min). Labs were significant for leukocytosis (11.9 k/uL; reference range: 5–10 k/uL), thrombocytopenia (30 k/uL; reference range: 140–400 k/uL), and D-dimer (>20 nanomoles/L; reference range: less than 1.37 nmol/L). Arterial blood gas showed pH 6.86 (reference range: 7.35–7.45), pCO2 56.9 mm of Hg (reference range: 35–45 mmHg), bicarb 10.3 mEq/L (reference range: 22–26 mEq/L), and lactate 15 mmol/L (reference range: 2–4 mmol/L). The patient was started on intravenous fluids and broad-spectrum antibiotics after obtaining blood cultures. The patient was intubated to secure airway and transferred to our ICU for possible therapeutic plasma exchange (TPE). In our hospital, the patient developed petechial rash, hematochezia, and hematemesis. Labs revealed coagulopathy, acute kidney injury, and elevated transaminases. In addition to giving supportive treatment, the infectious disease team was consulted for antibiotic recommendations. Initial differential was toxin-mediated gastroenteritis. We initiated on vancomycin, ceftriaxone, and metronidazole in addition to obtaining stool cultures and toxins for *E coli*, Shiga, and *C. difficile*. Clindamycin was continued for toxin-binding effects for 48 hours. A computed tomography (CT) scan of the abdomen and pelvis showed diffuse gastrointestinal wall edema, bibasilar pulmonary consolidations, distended endometrial cavity, small bilateral pleural effusions, and right adrenal gland nodular hyperplasia. Blood cultures, urine cultures, and stool studies were negative. The obstetrics and gynecology team performed transvaginal ultrasound to rule out endometritis or abscesses. No abscess or endometritis were found on ultrasound. After 2 days of admission, we received a report from the other hospital that blood cultures were positive for group B S*treptococcus* (*Streptococcus agalactiae*). Antibiotics were narrowed down to ceftriaxone and continued for a total of 14 days. Her hospital stay was also complicated by ST-elevation myocardial infarction (STEMI). The electrocardiogram showed ST segment elevations in leads I, II, aVL, V5, and V6 with reciprocal ST depressions in V1–V3. Troponin trended up to 37.60 ng/ml (reference range: below 0.04 ng/ml). Transthoracic echo showed left ventricular (LV) systolic dysfunction with an ejection fraction (EF) of 15–20%. The patient was started on dobutamine infusion for inotropic support. The patient was also started on continuous renal replacement therapy. Cardiology deferred any intervention since the patient was not hemodynamically stable. However, repeat echo showed improvement with an EF of 50% with mildly reduced LV systolic function. Dobutamine drip was eventually weaned, and the patient was extubated after 8 days.

## 3. Investigations

  Blood and stool cultures
  Transvaginal ultrasound
  CT abdomen and pelvis

## 4. Differential Diagnosis

  Toxin-mediated gastroenteritis

## 5. Treatment

Ceftriaxone IV for 2 weeks.

Therapeutic plasma exchange (TPE): it was not a recommended treatment for TSS. The therapeutic role of plasmapheresis in patients with toxic shock syndrome remains controversial. It was started initially when we did not have a definitive diagnosis as a supportive measure. The patient underwent a total of 5 rounds of therapeutic plasma exchange (TPE) during hospitalization because there was some evidence that TPE might be able to improve disseminated intravascular coagulation and septic cardiomyopathy in some patients [3].

## 6. Outcome and Follow-Up

Her renal function slowly improved, and metabolic acidosis was resolved. The infectious disease team also recommended administering *Hemophilus* B, influenza, Pneumovax, Prevnar, and meningococcal vaccines. The patient was transferred to a long-term acute care facility after 15 days of hospitalization in stable condition.

## 7. Discussion

GBS has become a global concern, with increasing incidence in the elderly, pregnant women, and neonates. Other populations at risk are patients with DM, malignancy, neurologic disorders, renal failure, cirrhosis, HIV infection, and IV catheterization and splenectomy patients. GBS can cause bacteremia, skin and soft tissue infections, UTI, peritonitis, pneumonia, and streptococcal shock-like syndrome [4].

GBS is not a common organism among the first differential diagnosis in nonpregnant population though the annual incidence of invasive GBS has been increasing in the past two decades, reaching 2.4–4.4 cases per 100,000 population. Previous surveillance studies have reported that immunocompromised conditions, diabetes mellitus, and malignancy were strongly linked with this invasive GBS [5]; however, splenectomy was not emphasized though it increases the risk to infections with encapsulated organisms.

In our literature review, we found a couple of case reports of invasive GBS in postsplenectomy patients. Campbell reported a case of GBS infection in a postsplenectomy patient due to liver cirrhosis presented with shortness of breath and developed hypotension and subsequently diagnosed with invasive GBS [6]. Chihara et al. reported a similar case of toxic shock syndrome (TSS) by group B S*treptococcus* (GBS) in an asplenic young woman, who presented with vomiting and diarrhea [7]. O. C. Finnegan et al reported a case of overwhelming post-splenectomy infection (OPSI) with GBS which occurred 10 years post splenectomy in an otherwise normal male adult [8].

Toxic shock-like syndrome is an acute, multisystem, toxin-mediated infection, resulting in shock and multiorgan dysfunction. Bacterial pyrogenic toxins play a key role in the pathogenesis of TSS. It was demonstrated that a small number of GBS strains can produce uncharacterized pyrogenic toxins which can cause toxic shock-like syndrome [9]. Streptococcal toxic shock syndrome was historically associated with group A streptococci; however, increasing evidence was found that other streptococci including groups B, C, or G can cause toxic shock-like syndrome [10]. GBS toxic shock-like syndrome is rare, and a few cases were reported in the literature [9, 11]. Our patient was found to have GBS bacteremia without a clear source, and the primary GBS bacteremia comprised 40% of invasive GBS cases [12]. Most GBS infections in adults may not have distinct features that would lead to suspect GBS infection; however, physicians should perform a detailed history and physical examination to find risk factors or the source of infection. The management of invasive GBS toxic shock-like syndrome requires a multidisciplinary approach with early supportive measures, antibiotics, and in some cases surgical intervention. In females, genital examination should be performed and if any tampon or foreign body is present that should be removed. Since there is no vaccine available against GBS, early antibiotic administration against GBS should be considered [13]. Physicians should consider invasive GBS infection as one of the differentials in splenectomy patients. A multivalent Ia, Ib, II, III, or V vaccine would provide immunity against 93% of serotypes causing invasive GBS infection in nonpregnant adults based on a data from Maryland [14]. The therapeutic role of plasmapheresis in patients with toxic shock syndrome remains controversial. We were able to find a few case reports that suggest plasmapheresis may have beneficial effects. The hypothesis behind that is plasmapheresis will remove streptococcal toxins and inflammatory mediators [15].

## 8. Conclusion

Invasive GBS infection can be complicated by streptococcal toxic shock-like syndrome (STLS), a condition characterized by hypotension often accompanied by fever or rash with rapid progression to shock and multiorgan failure as seen in our case. Early diagnosis and optimal antibiotics are crucial to achieving a successful outcome in these situations. It has been documented that 10% of GBS infections evolve to invasive disease, in which more than 25% will die within 24 hours of presentation [16]. There is a need for further research on GBS-preventive measures in adults with risk factors. Creating a vaccine against GBS is one of them, which is under process. A conjugate vaccine targeting the GBS capsule, as a major virulence factor, adding pilus protein surface antigens to trigger an immune response, can protect those adults at risk of invasive infection [17].

## Additional Points

(1) Encapsulated organisms such as *Streptococcus pneumoniae, Neisseria meningitides, and Haemophilus influenzae* are the leading causes of infection and increased mortality in postsplenectomy patients. (2) The incidence of invasive group B streptococcal disease (GBS) in nonpregnant adults is on the rise. (3) The first line of treatment choice for GBS is penicillin.
